# Specifying cellular context of transcription factor regulons for exploring context-specific gene regulation programs

**DOI:** 10.1093/nargab/lqae178

**Published:** 2025-01-07

**Authors:** Mariia Minaeva, Júlia Domingo, Philipp Rentzsch, Tuuli Lappalainen

**Affiliations:** Science for Life Laboratory, Department of Gene Technology, KTH Royal Institute of Technology, Tomtebodavägen 23A, 17165 Solna, Sweden; New York Genome Center, 101 Avenue of the Americas, New York, NY 10013, USA; Science for Life Laboratory, Department of Gene Technology, KTH Royal Institute of Technology, Tomtebodavägen 23A, 17165 Solna, Sweden; Science for Life Laboratory, Department of Gene Technology, KTH Royal Institute of Technology, Tomtebodavägen 23A, 17165 Solna, Sweden; New York Genome Center, 101 Avenue of the Americas, New York, NY 10013, USA

## Abstract

Understanding the role of transcription and transcription factors (TFs) in cellular identity and disease, such as cancer, is essential. However, comprehensive data resources for cell line-specific TF-to-target gene annotations are currently limited. To address this, we employed a straightforward method to define regulons that capture the cell-specific aspects of TF binding and transcript expression levels. By integrating cellular transcriptome and TF binding data, we generated regulons for 40 common cell lines comprising both proximal and distal cell line-specific regulatory events. Through systematic benchmarking involving TF knockout experiments, we demonstrated performance on par with state-of-the-art methods, with our method being easily applicable to other cell types of interest. We present case studies using three cancer single-cell datasets to showcase the utility of these cell-type-specific regulons in exploring transcriptional dysregulation. In summary, this study provides a valuable pipeline and a resource for systematically exploring cell line-specific transcriptional regulations, emphasizing the utility of network analysis in deciphering disease mechanisms.

## Graphical Abstract

**Graphical Abstract:**
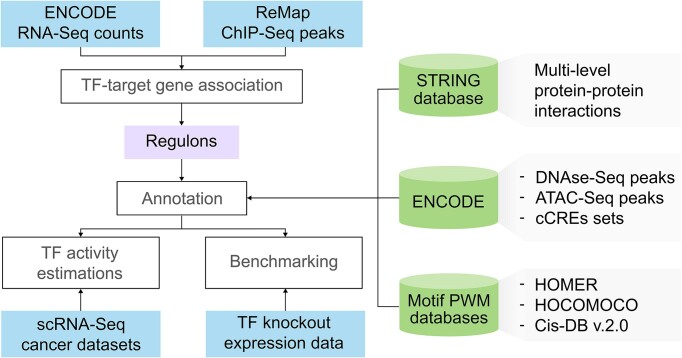

## Introduction

Transcriptional regulation plays a crucial role in cellular function, development (1,2), responses to environmental factors (3) and pathologies (4–7), including cancer (8,9). The human genome currently contains over 1600 annotated transcription factors (TFs), which are often tightly regulated and cell-type specific (10). This highlights the importance of investigating transcriptional regulation within specific cellular contexts. Extensive efforts have been directed towards understanding transcriptional regulation, resulting in the development of methods to construct regulons—sets of TF–target gene interactions that can be either direct or indirect. These methods vary in their data sources, curation levels and underlying hypotheses, lacking a unified annotation strategy.

One common method for creating regulons is manual literature curation. Several such databases like TRRUST (11), SIGNOR (12) and PAZAR (13) currently encompass regulons for ∼800 human TFs. While this approach provides highly confident annotations, it is resource-intensive and difficult to scale. To address this limitation, text-mining techniques have been employed (14). These methods assign confidence scores to sentences in abstracts of scientific articles. Highly confident sentences are either then directly used to construct regulons or aid in the more rapid manual curation process. This approach has led to the development of the CollecTri resource (15), which houses regulons for 1183 TFs and provides insights into regulatory interactions’ modes, such as activation or repression of target genes making it valuable for estimating TF activity from downstream gene expression. However, while the CollecTri strongly benefits from its scale and high confidence of included curated sources, it lacks cellular context annotations.

Data-driven methods complement literature-driven approaches by integrating cellular context into gene regulatory networks (GRNs). These methods are mainly divided into co-expression-based and TF-binding-based approaches. Co-expression methods analyze transcriptomic data to identify genes interacting with a specific TF based on shared expression patterns (16–18), but they suffer from high false discovery rates due to non-causal correlations in gene expression (19,20). In contrast, TF-binding methods utilize high-throughput chromatin immunoprecipitation followed by DNA sequencing (ChIP-Seq) data to identify TF-DNA binding events in promoter and enhancer regions. Databases like ChIP-Atlas (21) and GTRD (22) provide such data. However, these databases often lack consideration of cellular transcriptional profiles when constructing regulons. For instance, ChIP-Atlas generates regulons for specific cell lines, but it does not account for distinct gene expression patterns, potentially leading to associations with unexpressed genes in the studied cell line.

Given their partial overlap and complementary nature, various efforts have been made to integrate data-driven and manually curated databases to offer regulons at multiple confidence levels. For example, databases like DoRothEA (23) and CHEA3 (24), both combine ChIP-Seq and coexpression-derived networks. DoRothEA also includes motif-based predictions and literature-curated sources. Another database, RegNetwork (25), contains predicted, literature-curated and network-driven interactions, including protein–protein interactions (PPIs), for both target genes and target microRNAs. Despite their comprehensive coverage, these resources still have a notable number of false positive associations, primarily due to reliance on predictions, and they lack cell-type-specific information (15).

In this context, we present a straightforward method to define regulons that capture the cell-specific aspects of both TF binding and target gene expression. Our approach uses data from ChIP-Seq and RNA-Seq experiments to construct regulons and is adaptable to any cell type with such data (26,27). Here, we applied it to forty widely used cell lines with available ChIP-Seq data for a large number of TFs and functionally characterized the resulting regulons using various types of biological networks. To validate our approach, we systematically benchmarked our regulons against existing resources (CollecTri, DoRothEA, ChIP-Atlas, TRRUST, RegNetwork) using the KnockTF database (28), showing comparable performance with these methods. We also included various annotation levels supporting interactions that can be used to filter out false positive TF–target gene interactions. Through case studies, we demonstrated the ability of our regulons to identify relevant TF dysregulations in single-cell RNA-Seq datasets from three cancer types, underscoring the significance of cell-type-specific transcription studies. Our pipeline and the regulons are available open access.

## Materials and methods

### Data sources

Bulk RNA-Seq expression profiles of considered 40 cell lines were obtained from ENCODE ((26) and Supplementary Table S1). Transcript expression values were averaged for isogenic replicates. Non-redundant ChIP-Seq data were acquired from the publicly available ReMap v.2022 database (27) and underwent filtering to exclude regions present in the ENCODE blacklist (29). Additional K-562-specific ChIP-Seq peak data for the TF MYB were sourced from the GEO database (GSE124541). The original peak calling tool (30) was employed using the GRCh38 human genome assembly. Further GFI1B data for K-562 cells were retrieved from the GEO database (GSE117944; (22)), and a lift-over to the GRCh38 human genome assembly was performed. ATAC-Seq and DNase-Seq data were collected from ENCODE as NarrowPeaks files (Supplementary Tables S2 and S3). Candidate *cis-*regulatory element regions (cCREs) were downloaded from the SCREEN ENCODE platform (31).

### Mapping strategies

We introduced five distinct methodologies: ‘single TSS within 2 Mb’ (S2Mb), ‘single TSS within 100 kb’ (S100Kb), ‘single TSS within 2 Kb’ (S2Kb), ‘multiple TSS within 100 kb’ (M100Kb) and ‘multiple TSS within 2 kb’ (M2Kb), each characterized by different transcription start site (TSS) selection criteria and varying window sizes around them (Table 1). The choice of distance cutoff depends on the intended downstream application: larger windows can capture distal enhancers across the full *cis-*regulatory region but may also result in a higher number of false positive associations. The decision to focus on the top 50% of expressed transcripts was biologically motivated, as most genes typically exhibit one or a few highly expressed isoforms in a given cell type (32,33). Although somewhat arbitrary, this threshold helped eliminate isoforms that likely represent transcriptional or post-transcriptional noise, particularly in highly expressed genes (34).

**Table 1.** Overview of proposed approaches; here TSS stands for transcription start site

	Single TSS^1^ within 2 Mb [S2Mb]	Single TSS^1^ within 100 kb [S100Kb]	Single TSS^1^ within 2 kb [S2Kb]	Multiple TSS^1^ within 100 kb [M100Kb]	Multiple TSS^1^ within 2 kb [M2Kb]
**Distance to TSS**	[+1; −1] Mb	[+50; −50] kb	[+1; −1] kb	[+50; −50] kb	[+1; −1] kb
**Selected TSS**	Highest expressed transcript	Highest expressed transcript	Highest expressed transcript	Top 50% expressed transcript	Top 50% expressed transcript
**# transcripts per gene**	Single	Single	Single	Multiple	Multiple

^1^Here TSS stands for transcription start site.

The TSS coordinates, as well as additional transcript and gene level annotations for coding and non-coding Ensembl genes present in the bulk RNA-Seq profiles of the respective cell lines, were obtained using biomaRt v.2.48.3. and Ensembl release 109. Additionally, genes in the K-562 cell line were annotated using Ensembl releases 100 and 110 to analyze the robustness of regulons to TSS annotations. Within these methodologies, we selected either the TSS coordinate of the highest expressed isoform (S) or the TSS coordinates for the top 50% expressed isoforms (M).

To annotate TSSs of putative target genes with corresponding TF binding sites, we employed the bedtools v2.29.1 closest tool. Subsequently, distance filtering was applied as outlined in Table 1 to exclude unwanted interactions. This mapping process yielded a single association between a specific peak and its corresponding target gene for the S2Mb, S100Kb and S2Kb methods. In contrast, for the M100Kb and M2Kb approaches, multiple associations were maintained between a peak and a gene due to the resolved isoform structure with a single peak per transcript but several transcripts per gene.

### Target gene annotation and enrichment analysis

We employed various binary features to characterize target genes. To annotate the promoters of target genes with ATAC-Seq and DNase-Seq peaks within a 2000 bp distance threshold around the TSS, we used the GenomicRanges v.1.50.2 R package. We also classified considered TFBS into regions with promoter-like signatures (PLSs), proximal enhancer-like signatures (pELSs) and distal enhancer-like signatures (dELSs) by overlapping them with cell-line specific ENCODE cCREs collections using the GenomicRanges package. For the motif annotation, we obtained positional weight matrices (PWMs) from the HOMER database, HOCOMOCO v.11 (35) and CIS-BP built 2.00 (36). To annotate TF motifs, we employed the ‘annotatePeaks’ tool of the HOMER v.4.11 software package.

For network enrichment analysis, we employed separate logistic regression models for each binary characteristic (Table 2) with the glm function from the stats R package. This analysis considered presence in the regulon as the dependent variable, while gene expression and binary characteristics were included as covariates. As a random control, we performed a permutation test with 1000 permutations for the glm model fit on the S2Mb regulons using the permmodels function of the predictmeans R package (37). To obtain odds ratios for the shuffled networks, we averaged odds ratio estimates across permutations. The comparison of log2 odds ratio distributions was conducted using the Wilcoxon test with FDR correction.

**Table 2.** Overview of binary annotations of constructed regulons

Feature	Description
is_ppi	Whether there is a PPI between a gene and a given TF in the STRING database (interaction score > 0.15) (38)
is_coexpressed	Whether the co-expression of a gene with a TF is >0.6, corresponding to the 75th quantile of observed correlations
is_network	Whether there is an interaction between a gene and knockdown of a given TF in CRISPRi-derived networks from Morris *et al.*

### KnockTF benchmarking

We utilized the benchmarking tool provided by the decoupler package at https://decoupler-py.readthedocs.io/en/latest/notebooks/benchmark.html. In essence, this tool first calculates TF activities for TF knockout experiments using differential gene expression profiles from the KnockTF2 database ((28) and Figure 2A). By leveraging prior knowledge about the perturbed TFs in knockout experiments, it then evaluates the predictive capacity of the networks using the area under the receiver operating characteristic (AUROC) and precision-recall curve metrics (AUPRC). Given that the true positive class is confined to the perturbations covered in the database, the metrics are computed using the Monte-Carlo method. In each permutation, the negative and positive classes are balanced by randomly subsampling the former. For each specific cell line, we curated relevant knockout experiments from the KnockTF2 database, applying a filter to retain only high-quality experiments with a logFC of the perturbed TF < −1. GM-12878 was excluded from the analysis due to the lack of perturbational experiments. To ensure fairness in the comparison, we considered the intersection of sources (TFs) between the analyzed regulons and treated interactions in the CollecTri and DoRothEA regulons without prior knowledge of their mode of action (i.e. activator or repressor). The benchmarking pipeline was executed with default parameters.

### Single-cell RNA-seq datasets

We acquired preprocessed single-cell data from breast cancer ((39); GSE176078) and hepatoblastoma liver cancer ((40); GSE180665) studies. For the hepatoblastoma dataset, we selected hepatocytes and a subset of neoplastic cells to ensure an equal number of cells in each group. Within the breast cancer dataset, we designated epithelial cells labeled as LumA_SC and Basal_SC as cancer cells, while no_scTYPER_call cells were identified as healthy controls, following the original study’s annotations. Differential expression analysis was performed using the Wilcoxon test within the Scanpy package. In the case of the acute myeloid leukemia (AML) dataset (41), we reprocessed the results of the differential expression provided by the authors by replacing the MAST method (42) with the Wilcoxon test within the Scanpy package. We compared leukemic cells originating from cluster 6 with other leukemic progenitors. Additionally, we compared leukemic and healthy cells from cluster 6. Genes with FDR below 0.01 were selected as differentially expressed genes (DEGs), without applying a log-fold change (logFC) cutoff.

### Activity estimations

TF activities for each case study dataset were estimated using the univariate linear model (ulm) from the decoupler package, following the standard guidelines provided at https://decoupler-py.readthedocs.io/en/latest/notebooks/dorothea.html. Briefly, TF activities were calculated as t-values using linear regression. In this process, the expression values (specifically log fold changes from differential expression analysis) were regressed against the ‘TF profile’ (logFC ∼ activity * ‘TF profile’). Here, a profile is a signed adjacency matrix that represents TF–target gene interactions, where activating interactions are marked with a 1 and repressive interactions with a −1. When the regulatory mode of a TF was unknown, it was treated as an activator. Thus, for the ChIP-Seq-derived network, where interaction modes were unavailable, all TFs were considered activators. In contrast, the CollecTri regulon used signed interactions due to its better performance (15). Dysregulated TFs were identified with a *P*-value threshold of <0.01, without applying any additional *t*-value thresholds.

### Disease gene enrichment analysis and interpretation

The lists of activated TFs underwent enrichment analysis using the enrich tool from the gseapy package along with gene sets from DisGeNET (43), OMIM_Expanded (44) and KEGG_2021_Human (45). An additional set of COSMIC consensus genes (46) was obtained via a decoupler package (47).

## Results

### Collected data

**Figure 1.**
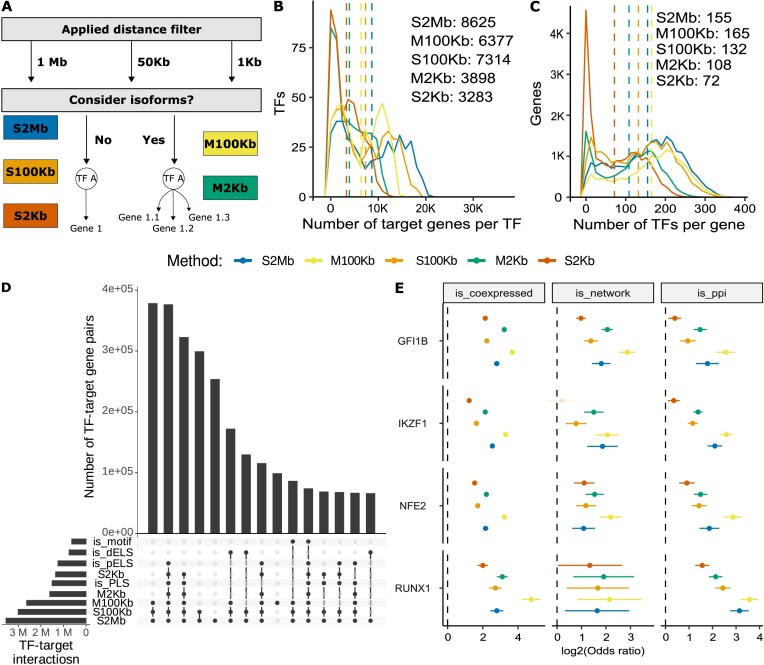
Overview of approaches and characteristics of K562 regulons. (**A**) Schematic overview of proposed methods with specified distance cutoff and number of considered transcripts; see Table 1 for abbreviations of the method names. (**B**) Distribution of the number of target genes per TF; dashed line shows per method median. (**C**) Distribution of number of TFs per target gene; dashed line shows per method median. (**D**) Overlap of regulons with TF binding motifs; the top 12 overlapping groups are shown. (**E**) Enrichment of regulons for K-562 cell line in biological networks: coexpression networks from bulk RNA-Seq data (left), trans-regulatory networks identified using STING-Seq technique (49) (middle), PPI networks (right). All, but S2Kb IKZF1 enrichment in trans-networks (shaded), are significant after the Benjamini-Hochberg adjustment (P < 0.05). TF denotes transcription factor; dELS and pELS stand for distal and proximal enhancer-like signatures respectively; PLS stands for promoter-like signature.

Cell type-specific RNA-Seq datasets are highly abundant, compared to other omics modalities. Thus, we developed a pipeline to associate TFs to their target genes at scale by leveraging cell-type specific RNA-seq datasets as well as TF binding data such as ChIP-Seq (see ‘Materials and methods’ section and Figure 1A). We constructed five TF–target gene sets (S2Mb, S100Kb, S2Kb, M100Kb and M2Kb), and hereafter, we refer to these target gene sets as regulons, while individual interactions are called TF–target gene interactions. Using K562 regulons as an example, the long-distance S2Mb, S100Kb and M100Kb approaches yielded more than double the number of target genes per TF compared to the S2Kb and M2Kb approaches (Figure 1B). The M2Kb approach identified ∼19% more target genes per TF than the most conservative S2Kb approach. The number of TFs targeting a gene in K562 varied from a median of 72 for S2Kb to 165 for M100Kb (Figure 1C). Similar patterns were observed for the three other cell lines with comprehensive data (Supplementary Figure S1 and Supplementary Table S4). We assessed the stability of the regulons with different TSS annotations and found almost perfect overlap among those constructed using TSS annotations from Ensembl releases 100, 109 and 110 (Supplementary Figure S2D).

### Characterization of TF–target gene interactions

To characterize our regulons, we overlapped them with known candidate cCREs from the ENCODE Project (31). Additionally, we annotated ChIP-Seq peaks associated with target genes with known TF binding motifs (TFBS) from HOMER (48), HOCOMOCO (35) and CIS-BP databases (36). Approximately 44% of TF–target gene interactions were exclusively identified by the long-distance approaches (S2Mb, S100Kb and M100Kb). Among those, 30% overlapped with dELSs suggesting that S2Mb, S100Kb and M100Kb can capture TF binding outside promoter regions (Figure 1D and Supplementary Figure S2). Approximately 17% of interactions displayed overlap with TFBS. Notably, for some TFs the lack of known PWMs complicated the motif annotation. Thus, employing a broader motif database could increase the number of confidently identified TF–target gene interactions and eliminate prior knowledge biases of these annotations.

Subsequently, we investigated the biological implications of the identified interactions by assessing their enrichment within various biological networks. Specifically, we examined co-expression networks, derived from ENCODE bulk RNA-Seq data (26), PPI networks sourced from the STRING database (38) and the trans-regulatory networks identified in CRISPRi experiments (49). Our results revealed a robust enrichment of our target genes in co-expression networks (Figure 1E and Supplementary Figure S3A) and PPIs (Figure 1E and Supplementary Figure S3B–E), often with higher values observed for the multiple isoform approaches (M100Kb and M2Kb) (Figure 1E and Supplementary Figure S3). Additionally, our K562 regulon was enriched in experimentally derived trans-regulatory networks for four tested TFs (GFI1B, NFE2, IKZF1 and RUNX1) (with log2 odds ratios ranging from 1 to 3), thereby reproducing previously observed results (49). These enrichments indicate that our TF regulons capture regulatory networks and benefit from considering multiple transcripts and longer distances.

### Benchmarking regulons using TF knockout experiments

**Figure 2.**
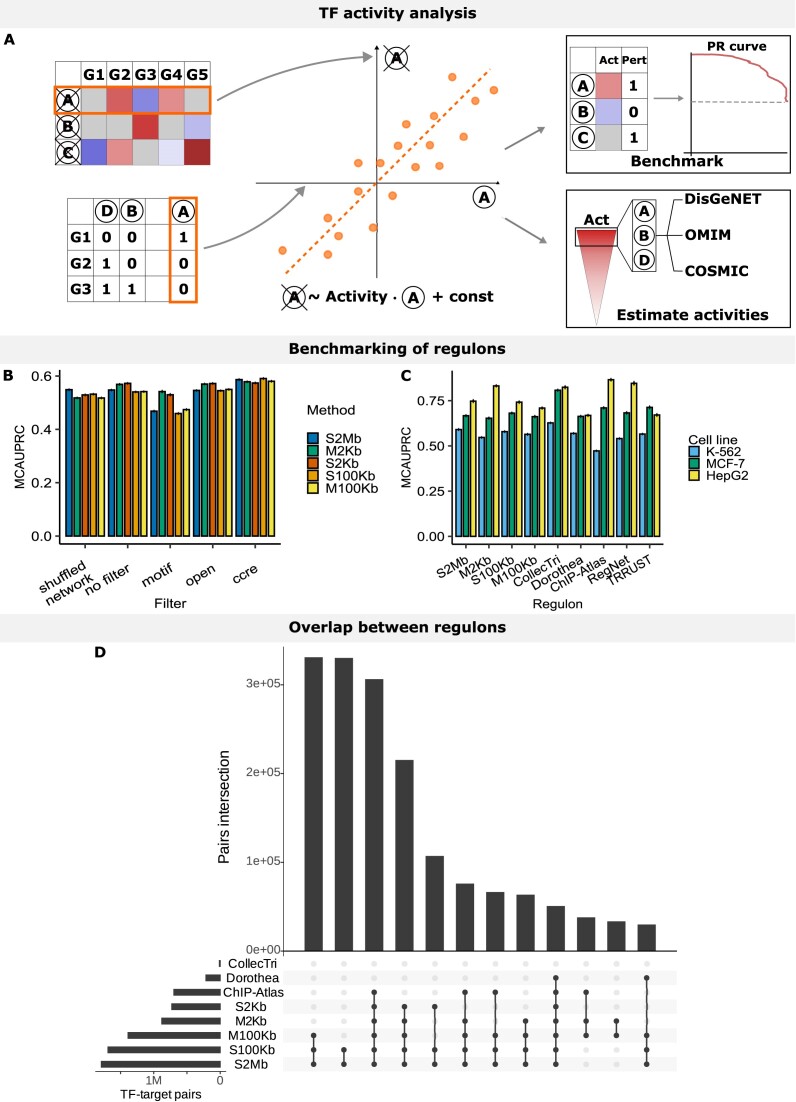
Benchmarking of the regulons. (**A**) Schematic overview of the benchmarking procedure. Here, expression values from a TF knockout experiment and a regulon of interest (left) are used as inputs for activity estimation. Here different TFs are encoded using letters (e.g. A, B, D) and genes are referred to as Gx. We fit a univariate linear model (ulm) to model gene expression as a function of TF regulations and estimate activities of TFs as regression coefficients of this model (middle). Then, in a benchmarking setting, we classify TFs into perturbed or non-perturbed and estimate their goodness (right upper) or, for the case studies, we perform an activity-based ranking of TFs and an enrichment analysis (right bottom). (**B** and **C**) Average MCAUPRC metric represents the predictive power of a TF activity-based classifier built upon different regulons. (**B**) Comparison of filtering strategies for S2Mb, S100Kb, S2Kb, M100Kb and M2Kb for K-562 regulons. (**C**) Comparison of regulons in identifying TF perturbations. (**D**) Overlap in the TF−target gene interactions between K-562 regulons from different resources; for ease of interpretation, the top 12 overlapping groups are shown. Here ‘Act’ stands for activity, ‘Pert’ stands for perturbations, ‘PR curve’ stands for precision-recall curve, and MCAUPRC stands for Monte-Carlo Area Under the Precision-Recall Curve.

Next, we evaluated the constructed regulons, starting by examining their overlap with comparable publicly available datasets: CollecTri, ChIP-Atlas and DoRothEA (Table 3). Across the four cell lines with abundant data (K-562, HepG2, MCF-7 and GM-12878), the highest degree of overlap was observed within our approaches and ChIP-Atlas regulons (∼45% of interactions; Figure 2D and Supplementary Figure S4A–C). Notably, DoRothEA and CollecTri regulons demonstrated limited agreement with other datasets (up to 15% of interactions). The CollecTri regulons had limited overlap with others, primarily attributed to regulons’ size being two orders of magnitude smaller compared to other databases (Figure 2D and Supplementary Figure S4). Altogether, most interactions were shared across at least two datasets.

**Table 3.** Resources of TF–gene interactions used for comparison

Database	Number of TFs	Number of interactions in regulons	Annotation process	CT^2^	Data source	Raw data sources
GTRD^1^	852 (regulons provided for 502 TFs)	256 331	TFBSs in the region [−1000,+500] nt around the TSS	−	ChIP-Seq, ChIP-exo, ChIP-nexus, MNase-seq, DNase-Seq, FAIRE-seq, ATAC-Seq, RNA-Seq	GEO, SRA, ENCODE, modENCODE
ChIP-Atlas	1807	K-562: 1 529 999 Hep-G2: 2 068 087 MCF-7: 639 027 GM-12878: 655 040	Peaks located around (±1, 5 or 10 kb) TSSs of RefSeq coding genes	+	ChIP-Seq, ATAC-Seq, DNase-Seq, Bisulfite-seq	NCBI SRA
DoRothEA	1541	1 076 628	Literature-curated resources; closest gene to TFBS from ChIP-Seq; TFBS predictions in promoters; co-expression networks	−	Literature, ChIP-Seq, TFBS motifs, RNA-Seq	Diverse; see (23)
CollecTri	1183	45 856	Multi-source text-mining-based dataset	−	Article abstracts	MedLine, GOA, IntAct, TRRUST, CytReg, GEREDB and SIGNOR
TRRUST	800	8444	Abstract-based text mining followed by manual curation	−	Article abstracts	MedLine
RegNetwork	1456	369 277	Interactions documented in TRED and KEGG; TFBS predictions in promoters; PPI pairs that contain at least one TF; miRNA	−	Literature, TFBS motifs, PPIs, miRNA	TRED, KEGG, JASPAR, TRANSFAC, BioGrid, IntAct, KEGG, STRING, HPRD, miRNA databases

^1^Database is cell-type specific, but target gene sets submitted to MSigDB are not

^2^CT refers to the cell-type specificity of a regulon

Regulatory TF−target gene interactions should generally manifest as changes in the expression of target genes following TF perturbation. Thus, we employed the decoupler GRN benchmarking tool and the KnockTF database (see ‘Materials and methods’ section and Figure 2A) to benchmark the regulons.

Analyzing our five different regulon construction approaches in three cell lines (K-562, Hep-G2 and MCF-7), we observed a statistically significant difference (*P* ≤ 0.01, Wilcoxon test with FDR correction) in Monte-Carlo Area Under the Precision-Recall Curve (MCAUPRC) across most cell lines and approaches, compared to the permuted network (Figure 2B; Supplementary Figures S5A and B, S6A−C and S7A−C). On average, the short-distance, and especially the M2Kb, approaches outperformed long-distance ones, suggesting the usefulness of the stricter distance filter in mitigating non-functional associations (Figure 2B; Supplementary Figures S6C and S7C). We then explored whether prior knowledge-based filtering of regulons would improve their predictive power. The inclusion of a cCRE filtering step enhanced the regulons’ ability to predict the upstream TF perturbations (overall *P* < 0.05, Wilcoxon two-sided test; Supplementary Figures S5−S7). Interestingly, in both MCF-7 and HepG2, but not K-562 cell lines, a motif filter significantly enhanced the performance (Figure 2B; Supplementary Figures S6C and S7C). Our results underscore the similar performance of the 2 kb methods, with the optimal outcomes achieved through the application of a cCRE filter. We proceeded with M2Kb, S100Kb, M100Kb and S2Mb regulons, incorporating cCRE filters, for subsequent comparisons with other datasets.

Following the same benchmarking procedure and comparing our approaches to TRRUST, DoRothEA, RegNet, CollecTri and ChIP-Atlas, we found that no single regulon consistently outperformed others in predicting TF perturbations. Performance metrics displayed notable variations within methods across different cell lines (Figure 2C). CollecTRI outperformed other regulons in K-562 and MCF-7 cells, with average MCAUPRC of 0.63 and 0.81, respectively (Supplementary Figures S5C and D, and S6D and E), attributed to its incorporation of interactions from text-mining and curated data sources. Interestingly, for Hep-G2 cells, ChIP-Atlas showed the highest score of 0.87 (Supplementary Figure S7D and E). In general, the performance of all regulons was better in the Hep-G2 cell line, where fewer knockout experiments were available, likely for TFs with straightforward effects on target genes. The M2Kb approach consistently demonstrated solid performance across cell lines, yielding average MCAUPRC scores of 0.55, 0.65 and 0.83 for K-562, MCF-7 and Hep-G2, respectively (Figure 2C; Supplementary Figures S5C and D, S6D and E, and S7D and E). Notably, ChIP-Atlas slightly outperformed our approaches in MCF-7 and Hep-G2 cells (MCAUPRC of 0.71 and 0.87, respectively), despite employing similar mapping strategies, possibly due to the additional filtering of low-quality targets incorporated in our benchmarking pipeline (see ‘Materials and methods’ section). In summary, while our approaches did not rank among the top performers, they consistently demonstrated competitive performance on par with other data-driven regulons that employ much more intensive text-mining and data curation compared to our straightforward, easily and broadly applicable approaches.

### Regulon annotations identify the disrupted activity of TFs in disease

**Figure 3.**
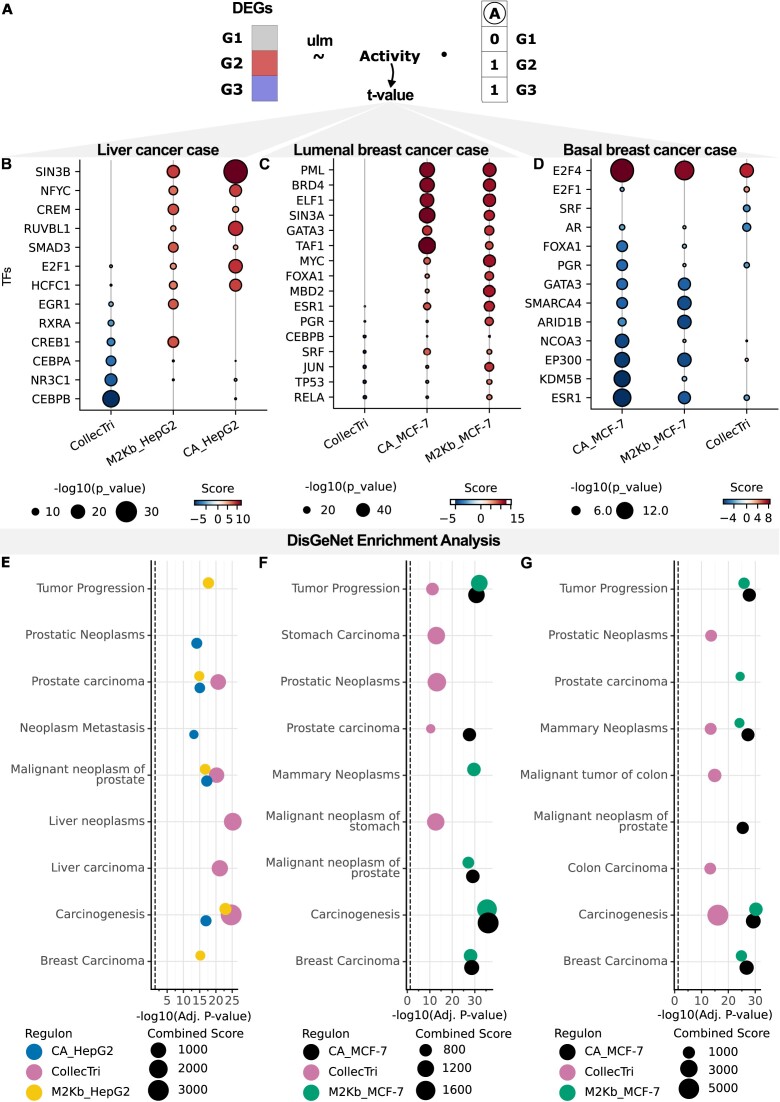
Case studies of detecting transcriptional dysregulation in cancers. (**A**) Schematic overview of the activity estimation procedure (see ‘Materials and methods’ section and Figure 2A). Here, ulm stands for a univariate linear model; DEG stands for differentially expressed genes. (**B**–**D**) Activity estimate for the top-ranked and biologically relevant TFs of each regulon for (**B**) hepatoblastoma (40), (**C**) lumenal type A breast cancer and (**D**) basal type breast cancer (39). Here, positive and negative activity scores mean activation and deactivation of TFs in malignant cells respectively. (**E**–**G**) Top 5 enriched terms of the enrichment analysis of dysregulated TFs in disease gene from DisGeNet database (43) for (**E**) hepatoblastoma, (**F**) lumenal type A breast cancer and (**G**) basal type breast cancer. Here ‘CA_cell line’ refers to ChIP-Atlas regulons for the specified cell line and ‘M2Kb_cell line’ refers to M2Kb regulons.

TFs can drive tumor growth and metastasis in a cancer-specific manner (50,51). Thus, we hypothesized that regulons tailored to specific cell types could capture disease-specific transcriptional patterns (52). As vignettes demonstrating potential applications of regulons, we utilized data from three single-cell RNA-Seq studies on distinct cancers: AML (41), breast cancer (39) and hepatoblastoma (40). By analyzing specific cell types in both healthy and diseased cells, we estimated TF activities (47) employing three cell-line-specific regulons for K-562, Hep-G2 and MCF-7 derived using the M2Kb approach and ChIP-Atlas, as well as a generalized CollecTri regulon (Figure 3A–D and Supplementary Figure S8A–C). Finally, we explored potential links between dysregulated TFs and cancer-associated genetic mutations through enrichment analysis using the databases DisGeNet (43), OMIM and COSMIC ((46); Figure 3E–G; Supplementary Figures S8D–E, S9 and S10).

First, using gene expression levels to infer TF activity similarly to the benchmarking analysis above, we compared transcriptional profiles between neoplastic and healthy liver cells (40) and then performed gene set enrichments of dysregulated TFs (Figure 3B and E). We observed that only CollecTri-detected dysregulated TFs were enriched in the gene sets associated with liver neoplasms in DisGeNet (Figure 3E). In contrast, the dysregulated TFs identified by both the ChIP-Atlas and M2Kb-based Hep-G2 regulons were enriched in broader cancer-related terms in DisGeNet, such as ‘Cancerogenesis,’ ‘Tumor Progression’ and ‘Neoplasm Metastasis’ (Figure 3E). Among the top activated TFs identified by both the M2Kb and the ChIP-Atlas regulons, five TFs (NFYC, RUVBL1, E2F1, HCFC1 and CREB1) have been classified as prognostic markers for lower patient survival in liver cancer ((53) and Figure 3B). Furthermore, we observed the activation of TFs associated with KEGG pathways such as ‘Transcriptional Misregulation in Cancer,’ ‘Hepatitis B’ and ‘Hepatocellular carcinoma’ (for both CollecTri and M2Kb) (Supplementary Tables S5 and S6).

We then explored the potential to differentiate cancer types by analyzing transcriptional dysregulations in malignant epithelial cells from luminal A and basal types of breast cancer (39). In luminal A breast cancer, dysregulated TF sets detected by both M2Kb and ChIP-Atlas-based MCF-7 regulons were significantly enriched in breast and prostate cancer terms in DisGeNet (Figure 3F; Supplementary Figures S9B and S10B). The CollecTri-based regulon identified a TF set enriched in prostatic and stomach carcinoma terms but not in breast cancer. Cell type-specific activity estimates (M2Kb and ChIP-Atlas) showed heightened GATA3 activity in luminal A breast cancer (Figure 3C), in contrast to reduced activity in basal type (Figure 3D), aligning with its known role in promoting luminal activity and cell differentiation (54). Furthermore, established marker genes of luminal A breast cancer, such as FOXA1 (55), ESR1 and PGR, were activated in malignant cells of the luminal A cancer type (Figure 3C). Conversely, all three regulon estimates indicated deactivation of most of these markers in basal-like cancer cells, consistent with the absence of ER, PR and HER2 markers in triple-negative breast cancer (Figure 3D).

Examining the transition from healthy-like leukemic hematopoietic stem cells (HSCs) to other abnormal AML progenitor cells (41), we discovered that differentially activated TFs exhibited enrichment in leukemia and cancer-related signatures (Supplementary Figure S8D) and are involved in hematopoiesis and AML-linked TFs, including GATA2 and SPI1 (56–58). Activation of MYC further supports increased stemness of healthy-like HSCs ((59) and Supplementary Figure S8B). We also compared leukemic activated HSCs to their dormant counterparts (41), identifying TFs essential for hematopoiesis and AML progression, such as MYC (60), SIN3A (61), SAP30 (62) and IRF1 ((63) and Supplementary Figure S8C). Altogether, we showcase how cell-type specific regulons could be used as a starting point for exploratory analysis of transcriptional dysregulations in specific conditions.

## Discussion

Transcription is pivotal in shaping cellular identity and pathology and has a complex multilevel regulation. Our focus lies on TFs, specifically delving into TF interactions with direct target genes through binding in their promoters or other *cis-*regulatory regions. Existing methods for building direct target regulons typically lack cell-type specificity or indirectly factor in cell-specific transcript expression. Furthermore, many of the more sophisticated approaches are complicated to implement and apply to new data sets and cell types of interest. To tackle this, we introduce a ChIP-Seq-based approach and data resource that provides straightforward and cell-type specific regulon annotation that includes an additional step to consider isoform-level gene expression.

In this study, we constructed regulons encompassing hundreds of TFs within forty frequently utilized cell lines. Analyzing four cell lines with the most data (K-562, MCF-7, HepG2 and GM12878), we showed enrichment of regulons in well-established biological networks and TFBS. To address the challenge of potential false positive interactions, we explored various filtering strategies based on external annotations and discerned that overlap with ENCODE cCRE annotations was advantageous.

The comparison of the proposed approaches with existing databases demonstrated a reasonable level of agreement, generally yielding comparable performance to similar data-driven regulons in predicting the effects of TF knockout. While our methods did not surpass state-of-the-art text-mining-based methods (15), the overall performance was similar. A distinguishing advantage of our approach lies in its simplicity and practical utility, relying solely on RNA-Seq and ChIP-Seq data. What differentiates our approach from other ChIP-Seq-based methods is the additional consideration of the transcriptional profile of potential target genes, which enhanced performance in specific cases (such as K-562) during benchmarking.

To exemplify the value of our regulons for context-specific exploration, we conducted case studies involving three cancer scRNA-Seq datasets. Our results illustrated the capacity of our regulons to identify well-known cancer-promoting regulatory programs, highlighting their ability to capture meaningful insights from complex biological data. However, it is important to note that the regulons we defined are derived from cancer cell lines, not primary cells from patients lacking the ChIP-seq and RNA-seq data needed to define regulons. While we do not claim that our regulons represent the true underlying networks of the respective cancers, they are a biological annotation layer that can help interpret transcriptome data and provide deeper insights into underlying biology compared to more generalized regulons.

However, it is important to acknowledge that the regulons constructed with our approach—as well as others—are likely to contain a significant number of false positive interactions, which can arise from various factors including the inherent noise in ChIP-Seq data (29,64,65) and nonfunctional TF binding that does not impact transcription. Potential false negatives stem from indirect binding and TF cooperation. Another notable consideration when relying on ChIP-Seq data is the limited coverage across cell lines, with additional biases toward well-studied and prominent TFs.

A key constraint of our proposed methods is that they link each potential TF binding site to the nearest gene. For the S2Kb and M2Kb approaches this limitation leads to the omission of longer-range interactions involving *cis-*regulatory elements farther away from the target gene. Although the S100Kb, M100Kb and S2Mb approaches can incorporate these interactions, they are likely to also capture false positive associations due to the possibility of a TSS of another gene being closer to the peak than the actual target. To circumvent both the arbitrary distance cutoff and associating a TFBS with a single nearest gene, an exponential decay model of TF binding could be applied (66). This model has been previously shown to be advantageous (67) over a cutoff approach utilized in this study. Additionally, regulons could be extended to incorporate TF-enhancer networks by using more sophisticated data or applying models to predict enhancer target genes, such as the ABC model (68).

Another important aspect is the consistency of regulons across approaches. Previous research indicates that regulons obtained through diverse methods exhibit limited overlap in identified interactions (14). Here, we similarly observed limited consistency between regulons derived from different methods. This might be attributed to several factors: (i) differences in interaction data sources, where some regulons utilise a single source while others incorporate multiple data sources and modalities, (ii) variations in cellular resolution between generalized and cell-type specific regulons, and (iii) exclusion or inclusion of indirect TF targets (Table 3). These differences indicate that the field is still far from converging into methods and resources with a high degree of agreement.

During this study, we employed a systematic benchmarking process to assess the quality and predictive power of the constructed regulons (28,69). This approach greatly facilitated comparability across different databases, yet it remains limited due to the relatively small number of covered TFs and cell lines (15). For example, in Hep-G2 cells, there were only three knockout experiments available for the 104 shared targets among all tested regulons in the KnockTF database (19). This sparsity of data might contribute to the considerable variation in the predictive capability of a single approach across various cell lines. None of the approaches clearly and consistently outperformed the others, and for the K-562 cell line with the most data, none of the methods showed high performance. While this benchmarking approach cannot be taken as the ground truth, these results indicate substantial room for development in TF regulon annotations.

Lastly, while our regulons proved valuable for identifying TF activity dysregulation in cancer, a notable drawback is their lack of information regarding the mode and quantitative strength of interactions, such as partial activation or repression. This introduces uncertainty into the sign of TF activity estimates and complicates the interpretation of observed dysregulations. The CollecTri regulons address this by distinguishing between activators and repressors, enhancing reliability for activity estimation. Nevertheless, quantitative assessment of TF regulatory strength remains a challenge and the data is often sparse. Since motif enrichment-based measures did not yield improved predictive power, an intriguing avenue for exploration could involve estimating these measures from the Hills equation based on co-expression networks or direct TF dosage-titration experiments (70,71).

In summary, we provided a straightforward approach for annotating TF regulons from cell-type specific TF binding and transcriptional profiles and applied it to forty different cell lines. We benchmarked the most abundant regulons against existing databases using TF knockout experiments and showcased their ability to identify cancer-related dysregulations, highlighting cases where cell type-specific regulons provided additional information compared to generalized approaches.

## Supplementary Material

lqae178_Supplemental_Files

## Data Availability

Raw and processed data files are available to download at https://doi.org/10.5281/zenodo.13861224. Accompanying code is available to review and download at https://github.com/LappalainenLab/chip_seq_regulons and https://doi.org/10.6084/m9.figshare.27153339.

## Notes

Present address: Mariia Minaeva, Institute of Computational Biology, Helmholtz Center Munich, Germany.

Present address: Júlia Domingo, Allostery Exploration Technologies, S.L., Barcelona 08003, Spain.
