# Supplementary material for: Specifying cellular context of transcription factor regulons for exploring context-specific gene regulation programs
Source: NAR Genom Bioinform. 2025 Jan 7;7(1):lqae178. doi: 10.1093/nargab/lqae178 (PMC11704787; doi:10.1093/nargab/lqae178)
Supplement: lqae178_Supplemental_Files [file lqae178_supplemental_files.zip › Minaeva et al. 2024 v4.0 Supplementary Data.pdf]

Supplementary data

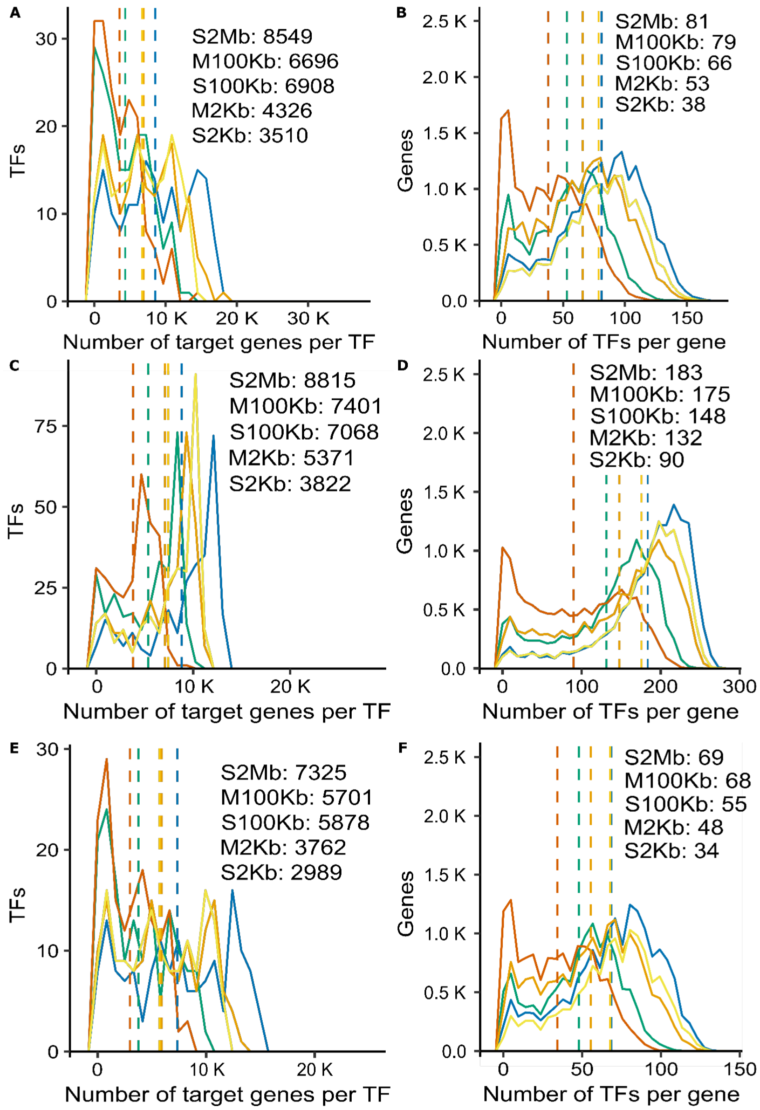

Figure S1: Characteristics of regulons, similar to Fig. 1B-C, for three additional cell lines: (A), (C), (E) Distributions of a number of target genes per TF; dashed line shows per method median. (B), (D), (F) Distribution of a number of TFs per target gene; dashed line shows per method median for (A) - (B) MCF-7 regulon, (C) - (D) Hep-G2 regulon, (E) - (F) GM-12878 regulon. S2Mb stands for "single TSS within 2 Mb"; M100Kb stands for "multiple TSS within 100 Kb"; S100Kb stands for "single TSS within 100 Kb"; M2Kb stands for "multiple TSS within 2 Kb"; S2Kb stands for "single TSS within 2 Kb".

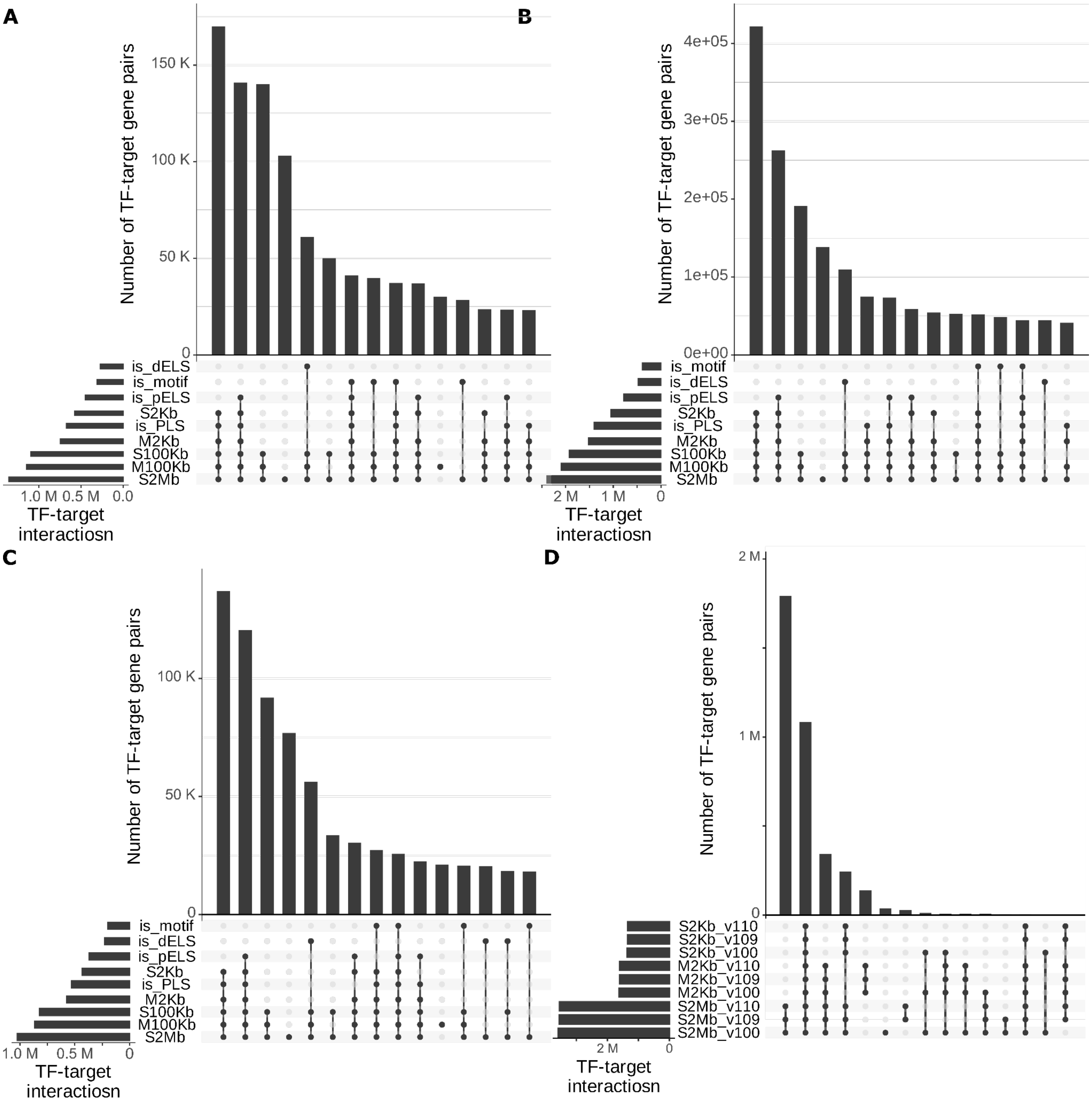

Figure S2: Overlap of regulons: (A) - (C) with TF binding motifs, similar to Fig. 1D. (A) MCF-7 cell line, (B) Hep-G2 cell line, (C) GM-12878 cell line; for ease of interpretation, the top 12 overlapping groups are shown. (D) Overlap of regulons for the K-562 cell line constructed using TSS annotations from multiple Ensembl releases. S2Mb stands for "single TSS within 2 Mb"; M100Kb stands for "multiple TSS within 100 Kb"; S100Kb stands for "single TSS within 100 Kb"; M2Kb stands for "multiple TSS within 2 Kb"; S2Kb stands for "single TSS within 2 Kb"; TF denoted transcription factor; dELS and pELS stand for distal and proximal enhancer-like signatures respectively; PLS stands for promoter-like signature.

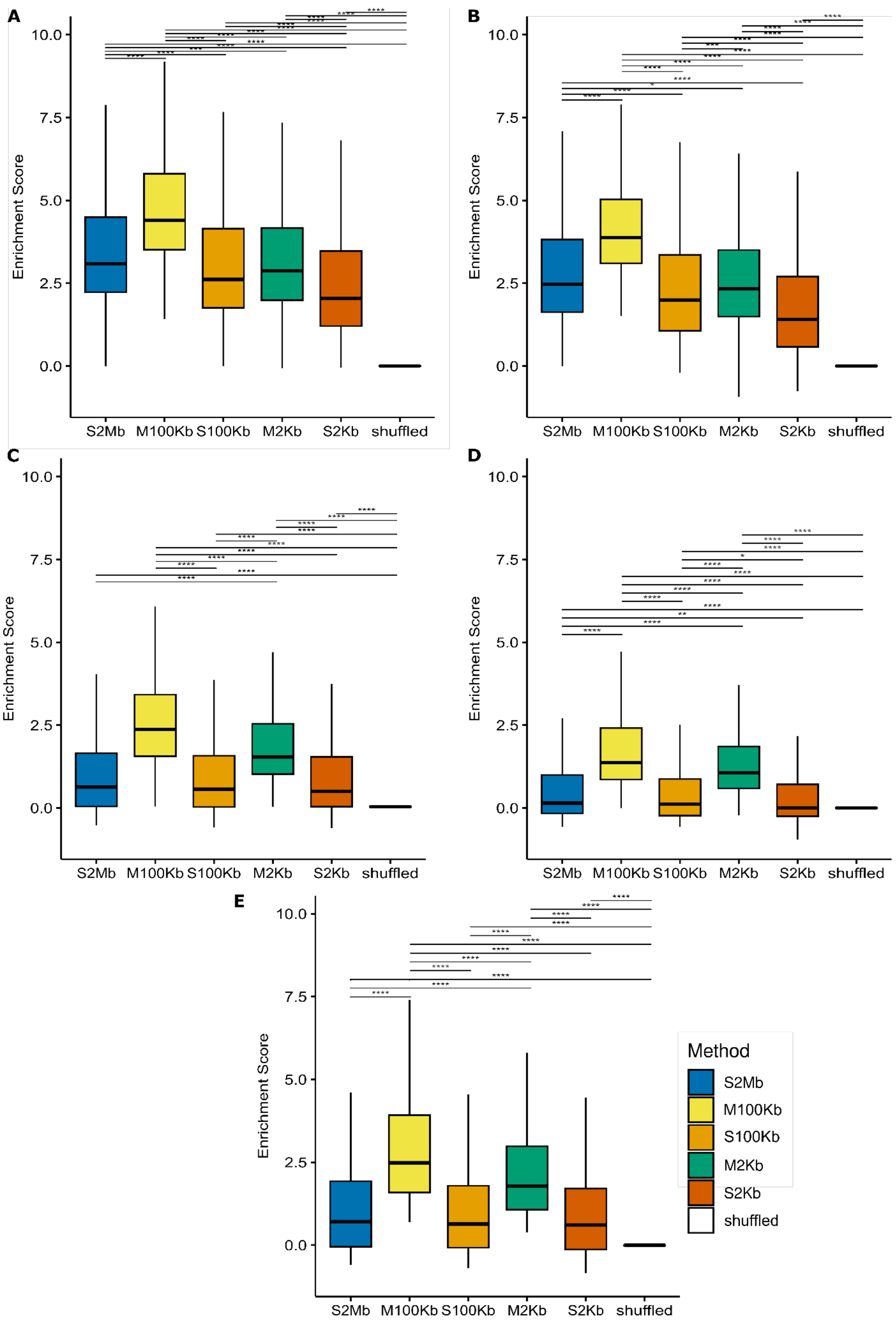

Figure S3: Enrichment of regulons in biological networks.(A) Enrichment of interactions from K-562 regulon in coexpression network derived from bulk RNA-Seq experiment (1). (B) - (E) Enrichment of interactions in protein-protein interaction networks from STRING (2) for (B) K-562, (C) MCF-7, (D) Hep-G2 and (E) GM-12878 regulons. Wilcoxon test with FDR correction is used to determine statistical significance. Asterisks: significant Benjamini-Hochberg–adjusted p-values (*P < 0.05, **P < 0.01, ***P < 0.001).

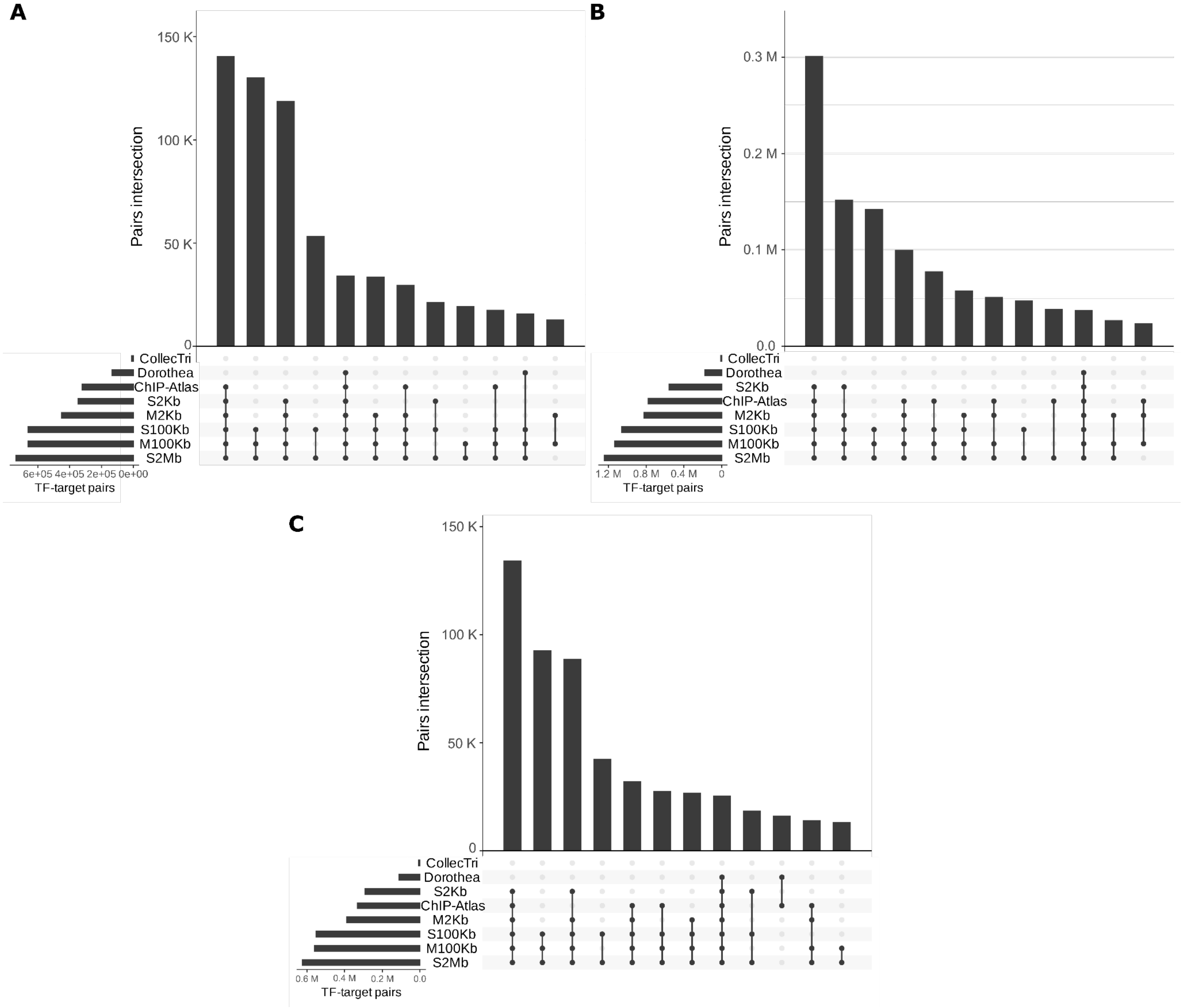

Figure S4: Comparison of different regulons, similar to Fig. 2D. Overlap in the TF-target gene pairs between regulons from different resources for (A) MCF-7, (B) Hep-G2 and (C) GM-12878 cell lines; for ease of interpretation, the top 12 overlapping groups are shown.

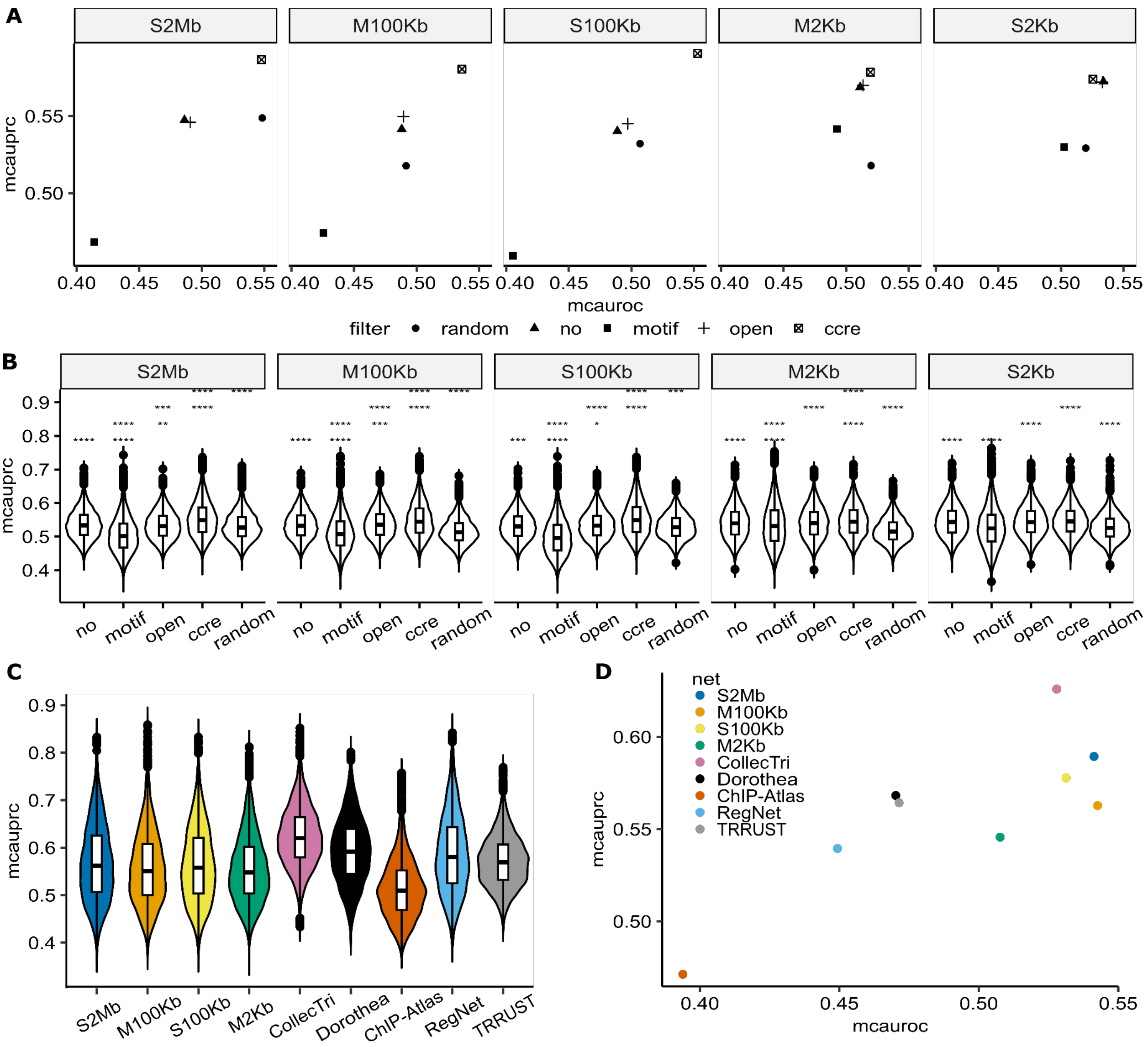

Figure S5: Benchmarking of K-562 regulon using knockout experiments from KnockTF database: (A) Impact of filtering strategy on regulon's performance in predicting TF knockout measured by mean MCAUROC (y-axis) and mean MCAUPRC (x-axis) for S2Mb (right), M2Kb (middle), and S2Kb (left) approaches. (B) Distribution of MCAUPRC for filtered regulons for S2Mb (right), M2Kb (middle), and S2Kb (left) approaches. (C) Comparison of the predictive power of regulons using MCAUPRC distribution. (D) Performance regulons in predicting TF knockout measured by mean MCAUROC (y-axis) and mean MCAUPRC (x-axis). MCAUPRC stands for Monte-Carlo Area Under the Precision-Recall Curve, MCAUROC stands for Monte-Carlo Area Under Receiver Operating characteristic Curve. Asterisks: significant Benjamini-Hochberg–adjusted p-values (*P < 0.05, **P < 0.01, ***P < 0.001).

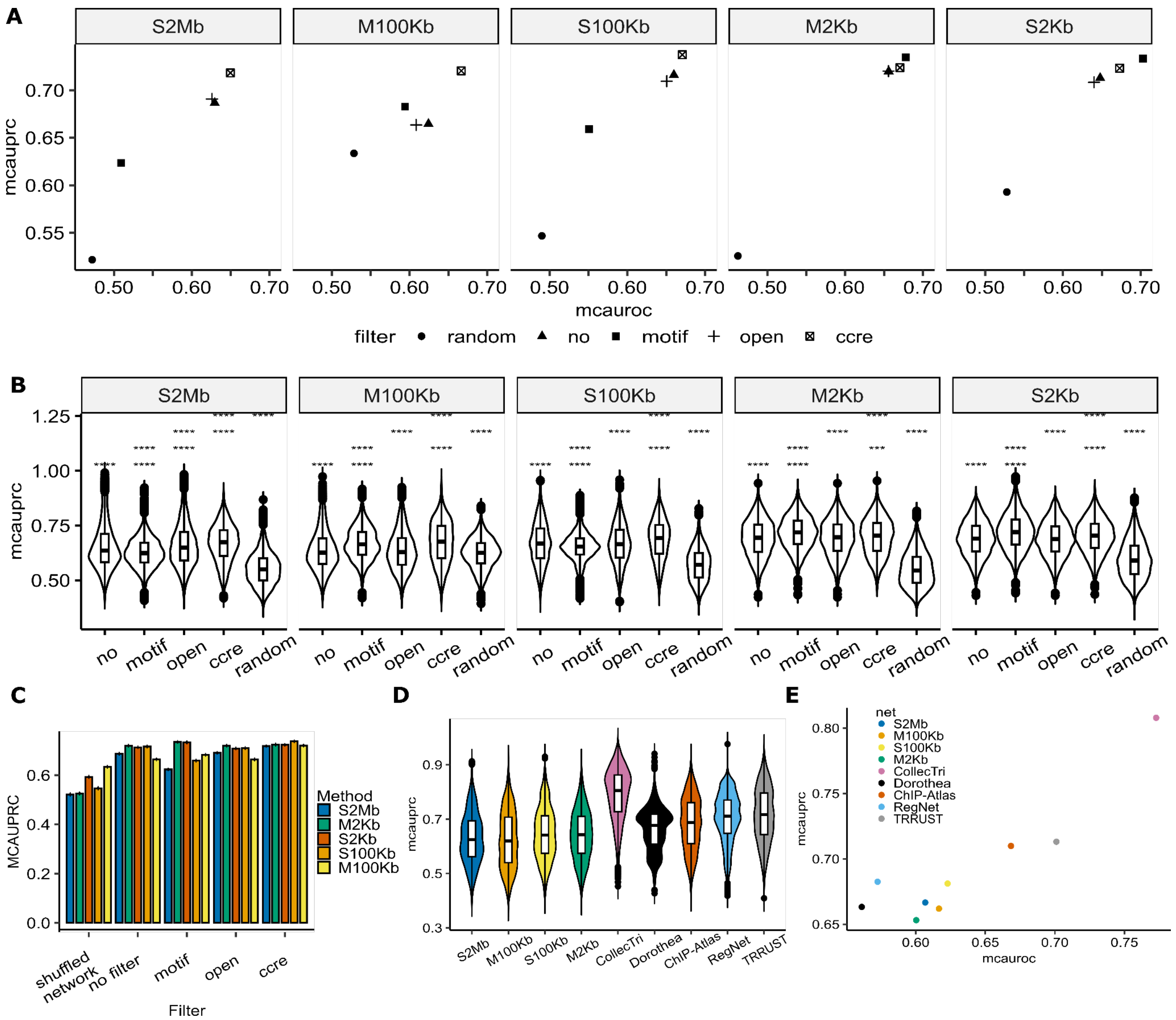

Figure S6: Benchmarking of MCF-7 regulon using knockout experiments from KnockTF database, similar to Fig. S5: (A) Impact of filtering strategy on regulon's performance in predicting TF knockout measured by mean MCAUROC (y-axis) and mean MCAUPRC (x-axis) for S2Mb (right), M2Kb (middle), and S2Kb (left) approaches. (B) Distribution of MCAUPRC for filtered regulons for S2Mb (right), M2Kb (middle), and S2Kb (left) approaches. (C) Comparison of filtering strategies for S2Mb (in blue), M2Kb (in red), and S2Kb (in green). (D) Comparison of the predictive power of regulons using MCAUPRC distribution. (E) Performance regulons in predicting TF knockout measured by mean MCAUROC (y-axis) and mean MCAUPRC (x-axis). MCAUPRC stands for Monte-Carlo Area Under the Precision-Recall Curve, MCAUROC stands for Monte-Carlo Area Under Receiver Operating characteristic Curve. Asterisks: significant Benjamini-Hochberg–adjusted p-values (*P < 0.05, **P < 0.01, ***P < 0.001).

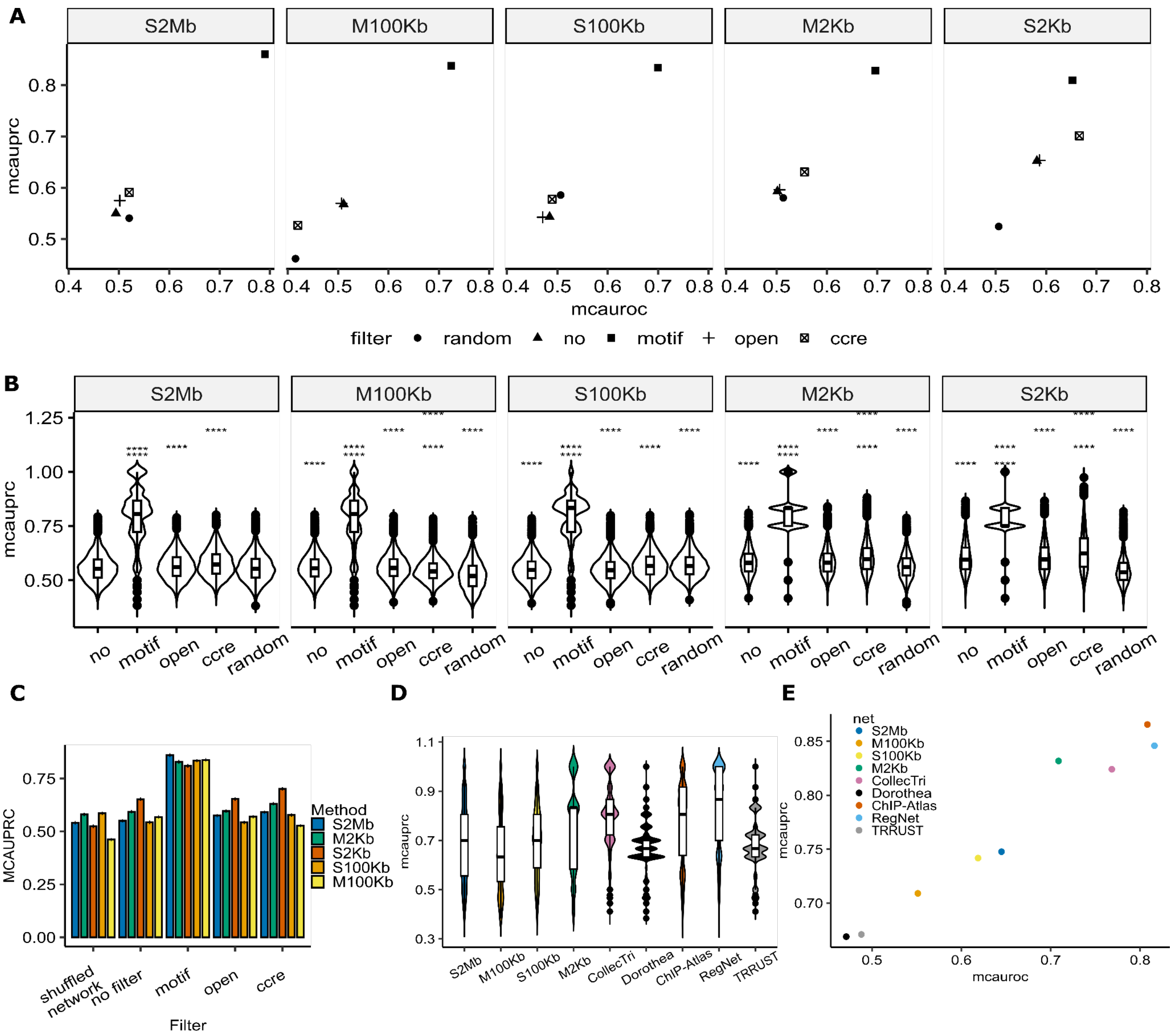

Figure S7: Benchmarking of Hep-G2 regulon using knockout experiments from KnockTF database, similar to Fig. S5: (A) Impact of filtering strategy on regulon's performance in predicting TF knockout measured by mean MCAUROC (y-axis) and mean MCAUPRC (x-axis) for S2Mb (right), M2Kb (middle), and S2Kb (left) approaches. (B) Distribution of MCAUPRC for filtered regulons for S2Mb (right), M2Kb (middle), and S2Kb (left) approaches. (C) Comparison of filtering strategies for S2Mb (in blue), M2Kb (in red), and S2Kb (in green). (D) Comparison of the predictive power of regulons using MCAUPRC distribution. (E) Performance regulons in predicting TF knockout measured by mean MCAUROC (y-axis) and mean MCAUPRC (x-axis). MCAUPRC stands for Monte-Carlo Area Under the Precision-Recall Curve, MCAUROC stands for Monte-Carlo Area Under Receiver Operating characteristic Curve. Asterisks: significant Benjamini-Hochberg–adjusted p-values (*P < 0.05, **P < 0.01, ***P < 0.001).

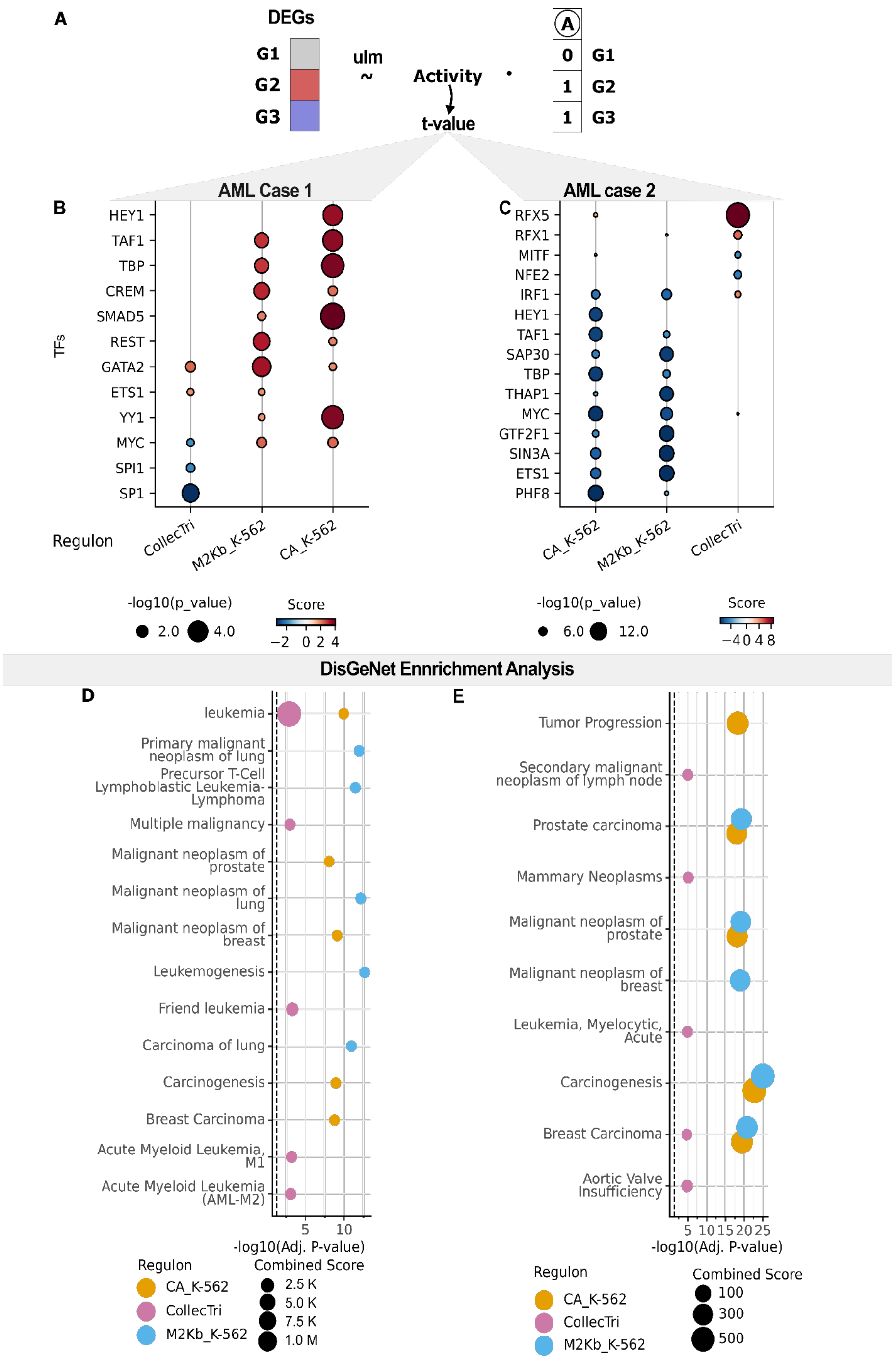

Figure S8: Case studies of detecting transcriptional dysregulation in acute myeloid leukemia, similar to Fig. 3. (A) Schematic overview of activity estimation procedure. (B) - (C) Activity estimate for the top-ranked and manually selected TFs of each regulon for (B) leukemic progenitor cells, and (C) hematopoietic stem cells (3). Here positive and negative activity scores mean activation and deactivation of TFs in malignant cells respectively. (D) - (E) Top 5 enriched terms of the enrichment analysis of dysregulated TFs in disease gene from DisGeNet database (4) for (D) leukemic progenitor cells, and (E) hematopoietic stem cells. “CA_cell line” refers to ChIP-Atlas regulons for the specified cell line and M2Kb_cell line” refers to M2Kb regulons. ulm stands for univariate linear model.

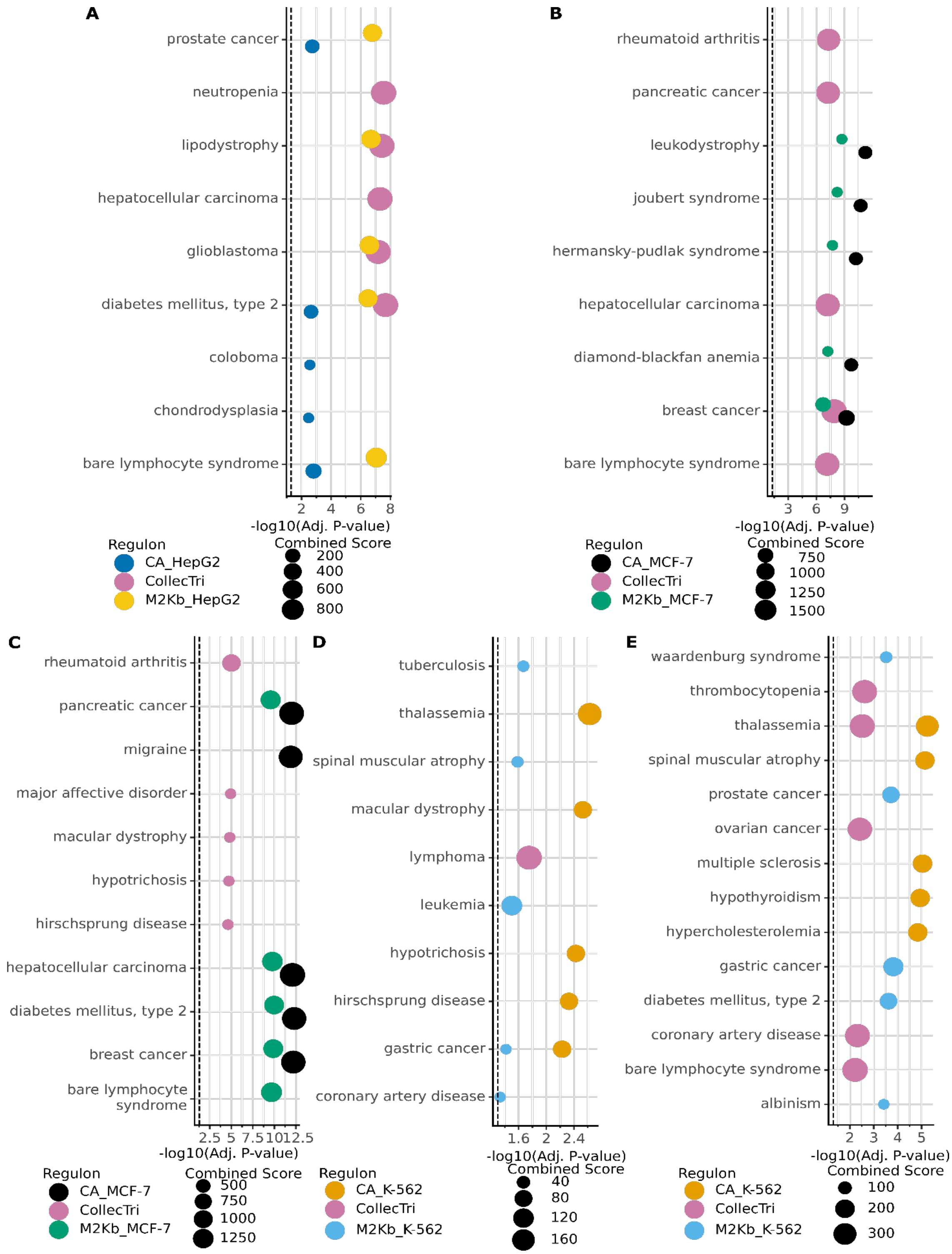

Figure S9: Enrichment analysis of dysregulated TFs in cancer versus disease genes from the OMIM database (5). (A) hepatoblastoma (6), (B) lumenal type A breast cancer (7), (C) basal type breast cancer (7), leukemic progenitors using K-562 (D), and (E) hematopoietic stem cells (3). “CA_cell line” refers to ChIP-Atlas regulons for the specified cell line and “cell line” refers to M2Kb regulons.

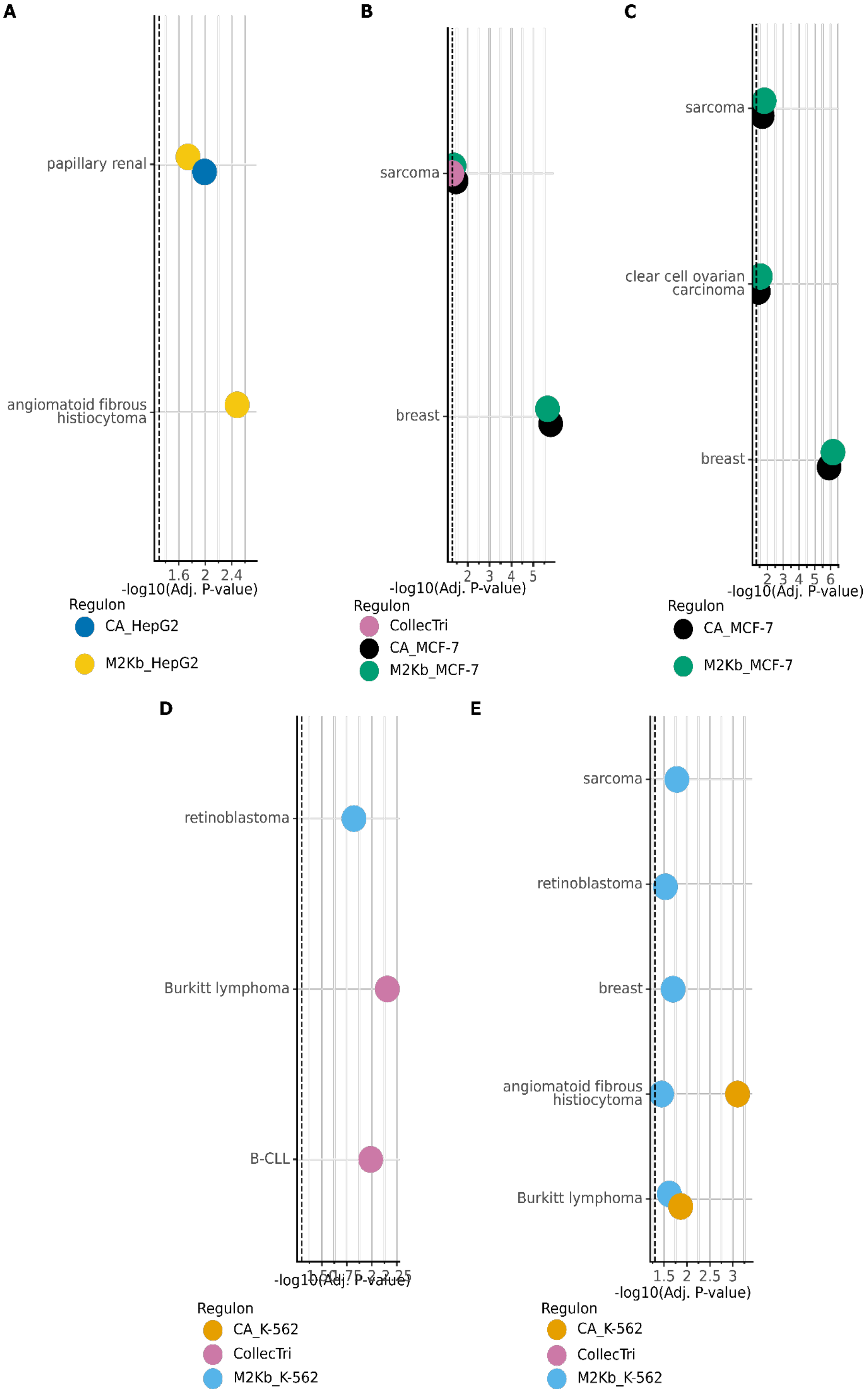

Figure S10: Enrichment analysis of dysregulated TFs in consensus gene from COSMIC database (8), similar to Fig. S9. (A) hepatoblastoma (6), (B) lumenal type A breast cancer (7), (C) basal type breast cancer (7), leukemic progenitors using K-562 (D), and (E) hematopoietic stem cells (3). “CA_cell line” refers to ChIP-Atlas regulons for the specified cell line and “cell line” refers to M2Kb regulons.

References:

1. Sloan,C.A., Chan,E.T., Davidson,J.M., Malladi,V.S., Strattan,J.S., Hitz,B.C., Gabdank,I., Narayanan,A.K., Ho,M., Lee,B.T., *et al.* (2016) ENCODE data at the ENCODE portal. *Nucleic Acids Res.*, **44**, D726–32.

2. von Mering,C., Jensen,L.J., Kuhn,M., Chaffron,S., Doerks,T., Krüger,B., Snel,B. and Bork,P. (2007) STRING 7--recent developments in the integration and prediction of protein interactions. *Nucleic Acids Res.*, **35**, D358–62.

3. Beneyto-Calabuig,S., Merbach,A.K., Kniffka,J.-A., Antes,M., Szu-Tu,C., Rohde,C., Waclawiczek,A., Stelmach,P., Gräßle,S., Pervan,P., *et al.* (2023) Clonally resolved single-cell multi-omics identifies routes of cellular differentiation in acute myeloid leukemia. *Cell Stem Cell*, **30**, 706–721.e8.

4. Piñero,J., Bravo,À., Queralt-Rosinach,N., Gutiérrez-Sacristán,A., Deu-Pons,J., Centeno,E., García-García,J., Sanz,F. and Furlong,L.I. (2017) DisGeNET: a comprehensive platform integrating information on human disease-associated genes and variants. *Nucleic Acids Res.*, **45**, D833–D839.

5. Hamosh,A., Scott,A.F., Amberger,J.S., Bocchini,C.A. and McKusick,V.A. (2005) Online Mendelian Inheritance in Man (OMIM), a knowledgebase of human genes and genetic disorders. *Nucleic Acids Res.*, **33**, D514–7.

6. Bondoc,A., Glaser,K., Jin,K., Lake,C., Cairo,S., Geller,J., Tiao,G. and Aronow,B. (2021) Identification of distinct tumor cell populations and key genetic mechanisms through single cell sequencing in hepatoblastoma. *Commun Biol*, **4**, 1049.

7. Wu,S.Z., Al-Eryani,G., Roden,D.L., Junankar,S., Harvey,K., Andersson,A., Thennavan,A., Wang,C., Torpy,J.R., Bartonicek,N., *et al.* (2021) A single-cell and spatially resolved atlas of human breast cancers. *Nat. Genet.*, **53**, 1334–1347.

8. Alsulami,A.F., Torres,P.H.M., Moghul,I., Arif,S.M., Chaplin,A.K., Vedithi,S.C. and Blundell,T.L. (2021) COSMIC Cancer Gene Census 3D database: understanding the impacts of mutations on cancer targets. *Brief. Bioinform.*, **22**, bbab220.
